# Inhibitory effect of *Clitoria ternatea* flower petal extract on fructose-induced protein glycation and oxidation-dependent damages to albumin *in vitro*

**DOI:** 10.1186/s12906-015-0546-2

**Published:** 2015-02-18

**Authors:** Poramin Chayaratanasin, Manuel Alejandro Barbieri, Nipattra Suanpairintr, Sirichai Adisakwattana

**Affiliations:** Department of Pharmacology, Faculty of Veterinary Science, Chulalongkorn University, Bangkok, 10330 Thailand; Program in Veterinary Biosciences, Faculty of Veterinary Science, Chulalongkorn University, Bangkok, 10330 Thailand; Department of Biological Sciences, Florida International University, Miami, FL 33199 USA; Biomolecular Sciences Institute, Florida International University, Miami, FL 33199 USA; Fairchild Tropical Botanic Garden, 10901 Old Cutler Road, Coral Gables, FL 33156 USA; Research Group of Herbal Medicine for Prevention and Therapeutic of Metabolic Diseases, Chulalongkorn University, Bangkok, 10330 Thailand; Department of Nutrition and Dietetics, Faculty of Allied Health Sciences, Chulalongkorn University, Bangkok, 10330 Thailand

**Keywords:** Antiglycation, Antioxidant, *Clitoria ternatea* flower extract, Anthocyanin, Fructose

## Abstract

**Background:**

The accumulation of advanced glycation end products (AGEs) in body tissue has been implicated in the progression of age-related diseases. Inhibition of AGE formation is the imperative approach for alleviating diabetic complications. *Clitoria ternatea* extract (CTE) has been demonstrated to possess anti-diabetic activity. However, there is no scientific evidence supporting its anti-glycation activity. The objective of this study was to determine the inhibitory effect of CTE on fructose-induced formation of AGEs and protein oxidation. Antioxidant activity of CTE was also assessed by various methods.

**Methods:**

The aqueous extract of CTE (0.25-1.00 mg/ml) was measured for the content of total phenolic compounds, flavonoid, and anthocyanin by Folin-Ciocalteu assay, AlCl_3_ colorimetric method, and pH differential method, respectively. The various concentrations of CTE were incubated with BSA and fructose at 37°C for 28 days. The formation of fluorescent AGEs, the level of fructosamine, protein carbonyl content, and thiol group were measured. The *in vitro* antioxidant activity was measured by the 1,1-diphenyl 2-picrylhydrazyl (DPPH) scavenging activity, trolox equivalent antioxidant capacity (TEAC), ferric reducing antioxidant power (FRAP), hydroxyl radical scavenging activity (HRSA), superoxide radical scavenging activity (SRSA), and ferrous ion chelating power (FICP).

**Results:**

The results demonstrated that the content of total phenolics, flavonoids and total anthocyanins in CTE was 53 ± 0.34 mg gallic acid equivalents/g dried extract, 11.2 ± 0.33 mg catechin equivalents/g dried extract, and 1.46 ± 0.04 mg cyanidin-3-glucoside equivalents/g dried extract, respectively. Moreover, CTE (0.25-1.00 mg/ml) significantly inhibited the formation of AGEs in a concentration-dependent manner. CTE also markedly reduced the levels of fructosamine and the oxidation of protein by decreasing protein carbonyl content and preventing free thiol depletion. In the DPPH radical scavenging activity and SRSA, CTE had the IC_50_ values of 0.47 ± 0.01 mg/ml and 0.58 ± 0.04 mg/ml. Furthermore, the FRAP and TEAC values of CTE were 0.38 ± 0.01 mmol FeSO_4_ equivalents/mg dried extract and 0.17 ± 0.01 mg trolox equivalents/mg dried extract. However, CTE showed weak scavenging activity on hydroxyl radical and a weak antioxidant iron chelator.

**Conclusions:**

The results showed that CTE has strong antiglycation and antioxidant properties and might have therapeutic potentials in the prevention of AGE-mediated diabetic complications.

## Background

Diabetes Mellitus (DM) is a group of metabolic diseases characterized by hyperglycemia, dyslipidemia, and abnormal protein metabolism that result from defects in both insulin secretion and/or insulin action. Chronic hyperglycemia is a major cause of complications of diabetes through 5 major mechanisms including polyol pathway, the formation of advanced glycation end products (AGEs), increased expression of AGEs receptor, Protein kinase C isoform activation and hexosamine pathway [1,2]. In general, non-enzymatic glycation is a complex series of reactions between the carbonyl group of reducing sugars (glucose, fructose, and ribose) and the amino group of proteins. Consequently, a reversible structure called as an unstable Schiff’s base is formed and spontaneously rearranged into an Amadori product such as fructosamine. During the propagation reaction, the Amadori products react with the amino acids to form irreversible AGEs, including fluorescent and crosslinking AGEs (such as pentosidine and imidazolones) and non-fluorescent and non-crosslinking AGEs (such as N^ε^-CML) [3-5]. The accumulation of AGEs in living organisms also contributes to functional modifications of tissue proteins, resulting in the progress of normal aging and the pathogenesis of age-related diseases, such as diabetes, cardiovascular diseases, and Alzheimer’s disease [6-8]. Fructose is one of the most common reducing monosaccharides found in blood circulation. Evidence supports that high fructose overconsumption has been associated with an increased risk of developing long-term diabetic complications [9,10]. Intracellular fructose is increased in a number of tissues in diabetic patients *via* the polyol pathway, resulting in glycation production approximately 10 times faster than glucose [11]. Therefore, there has been serious concern regarding the critical role of dietary fructose in metabolic diseases. Scientists are developing an alternative approach to preventing progression of diabetic complications through the reduction of AGE formation. Aminoguanidine (AG), a well-known antiglycating agent, inhibits the formation of AGEs and prevents the development of diabetic complications in animal models of diabetes. Nevertheless, aminoguanidine has been terminated due to serious adverse effects such as myocardial infarction, congestive heart failure, atrial fibrillation, anemia, and gastrointestinal disturbance [12,13]. There has been a great deal of interest in using plant-based foods for prevention and amelioration of AGE-mediated diabetic complications [4,14,15].

*Clitoria ternatea* L. (Family: Fabaceae) commonly known as butterfly pea has been used as a traditional Ayurvedic medicine as a memory enhancer, antistress, anxiolytic, antidepressant, anticonvulsant, tranquilizing, and sedative agent [14]. Its flower petal containing dietary anthocyanins is used as a natural blue colorant in a variety of foods. The extract of *Clitoria ternatea* possesses a wide range of pharmacological activities including anti-oxidant, antimicrobial, anti-inflammatory, antipyretic, anti-helminthic, and analgesic activities [16,17]. In addition, aqueous extract of *Clitoria ternatea* flower exerts anti-hyperglycemic effects in alloxan-induced diabetic rats [18]. To the best of our knowledge, there have been no previous studies that address the effect of *Clitoria ternatea* extract (CTE) on the inhibition of AGE formation. Therefore, the objective of the present study was to investigate the effect of CTE against bovine serum albumin (BSA) in fructose-induced non-enzymatic glycation. The study also examined the effect of CTE on glycation-induced protein oxidative damages. Antioxidant activity of CTE was also determined in various *in vitro* models.

## Methods

### Chemicals

Bovine serum albumin (BSA), aminoguanidine (AG), 2,2-diphenyl-1-picrylhydrazyl (DPPH), 2,2′-azino-bis(3-ethylbenzothiazoline-6-sulfonic acid) (Trolox), 2,4,6- tripyridyl-S-triazine (TPTZ), iron (II) sulfate (FeSO_4_), xanthine, xanthine oxidase, 5,5′-dithiobisnitro benzoic acid (DTNB), nitroblue tetrazolium (NBT), 1-deoxy-1-morpholinofructose (DMF), 2,4-dinitrophenylhydrazine (DNPH), thioflavin T reagent (4-(3,6-dimethyl-1,3-benzothiazol-3-ium-2-yl)-N,N-dimethylaniline chloride), and L-cysteine were purchased from Sigma Chemical Co. (St. Louis, MO, USA). Fructose, Folin-Ciocalteu’s phenol reagent, and gallic acid were purchased from Fluka (St. Louis, MO, USA). All other chemical reagents used in this study were of analytical grade.

### Plant materials

The dried flower of *Clitoria ternatea* was purchased from the local herbal shop in Bangkok, Thailand. The plant has been authenticated at the Princess Sirindhorn Plant Herbarium, Plant Varieties Protection Division, Department of Agriculture, Thailand, Voucher specimen: BKU066793. The extraction of the plant was modified according to a previously published method [19]. Briefly, the dried plant (300 g) was extracted with distilled water (1 L) at 95°C for 2 h. The sample was filtered through Whatman 70 mm filter paper. The aqueous solution was dried using a spray dryer SD-100 (Eyela world, Tokyo Rikakikai Co., LTD, Japan). The spray drying condition was inlet temperature (178°C), outlet temperature (80°C), blower (0.9 m^3^/min) and atomizing (90 kPa).

### The phytochemical analysis

The CTE was dissolved in distilled water before use. For measurement of total phenolic content, the sample was mixed with Folin-Ciocalteu reagent (previously diluted 10-fold with distilled water), followed by 2% Na_2_CO_3_ and kept for 2 h at room temperature. The absorbance of mixture was measured at the wavelength 760 nm with a spectrophotometer. Total phenolic content was expressed as mg gallic acid equivalents/g dry weight of extract [19]. Quantification of flavonoid constituents was performed according to a previously published method [19]. The extract was dissolved in 95% ethanol and 10% aluminum chloride, 1 M potassium acetate and distilled water, then kept at room temperature for 30 min. The mixture was measured the absorbance at the wavelength 415 nm with a spectrophotometer. Catechin was used as the standard. Total anthocyanin content (TAC) in the extract was determined by using pH differential method. The extract was added to two buffer systems including 0.025 M potassium chloride at pH 1.0 and 0.4 M sodium acetate at pH 4.5, respectively. The calculated absorption was determined using the equation of A = (A_510_ - A_700_) pH1.0 - (A_510_ - A_700_) pH4.5 and the TAC in the testing solution was calculated as cyanidin-3-glucoside equivalents [20].

### Antiglycation activities

The glycated BSA formation was performed according to a previously published method [21]. In brief, BSA (10 mg/ml) was incubated with 0.5 M fructose in 0.1 M phosphate buffered saline (PBS), pH 7.4 containing 0.02% sodium azide in the dark at 37°C for 7, 14, 21, and 28 days. Before incubation, CTE (0.25-1.00 mg/ml) and aminoguanidine (1.00 mM) were dissolved in PBS and added to the mixtures. The fluorescent AGE formation of glycated BSA was determined using a spectrofluorometer at excitation and emission wavelength of 355 nm and 460 nm, respectively. Aminoguanidine (AG) was used as a positive control in this study. The results were expressed as a percentage inhibition of the corresponding control values.

### Determination of fructosamine

After incubation for 7, 14, 21, and 28 days, the concentration of fructosamine was determined using a nitroblue tetrazolium (NBT) assay according to a previous study [21]. In brief, the glycated BSA was incubated with 0.5 mM NBT in 2 M sodium carbonate buffer (pH 10.4) at 37°C for 10 and 15 min time points. The absorbance was measured at the wavelength of 590 nm. The concentration of fructosamine (mg/ml) was calculated from the standard curve using 1-deoxy-1-morpholino-fructose (1-DMF).

### Determination of protein carbonyl content

After incubation for 7, 14, 21, and 28 days, the protein carbonyl content was determined using 2,4-dinitrophenylhydrazine (DNPH) according to a previous study [21]. Briefly, the glycated BSA was incubated with 10 mM DNPH in 2.5 M HCl in a dark room for 1 h. Protein precipitation was done by 20% w/v trichloroacetic acid (TCA) on ice for 5 min and centrifuged at 10,000 rpm at 4°C for 10 min. The protein pellet was washed with 0.5 ml of ethanol/ethyl acetate mixture (1:1 v/v) three times and then dissolved in 6 M of guanidine hydrochloride (pH 2.3). The absorbance was measured at wavelength of 370 nm. The carbonyl content was calculated from the extinction coefficient for DNPH (ε = 22,000 M^−1^ · cm^−1^). The results were expressed as nmol carbonyls/mg protein.

### Determination of protein thiol group

After incubation for 7, 14, 21, and 28 days, the free thiol concentration of glycated BSA was measured using Ellman’s assay [21]. Briefly, the glycated samples were incubated with 2.5 mM DTNB solutions for 15 min and then the absorbance was read at wavelength of 410 nm. The free thiol concentration of samples was calculated based on the standard curve prepared by using various concentration of L-cysteine.

### DPPH radical scavenging activity

DPPH (1,1-diphenyl 2-picrylhydrazyl) radical scavenging activity was measured according to the previous method [22]. Briefly, the extract was added with 0.2 mM DPPH as the free radical source and incubated for 30 min at room temperature. The decrease in the solution absorbance was measured at 515 nm. The IC_50_ value was calculated from plots of log concentration of inhibitor concentration versus percentage inhibition curves. Ascorbic acid was used as a positive control for this study.

### Trolox equivalent antioxidant capacity assay (TEAC)

Assessment of ABTS radical-scavenging activity was done according to a previously published method [22]. The radical anion (ABTS°^+^) was induced by adding potassium persulfate (K_2_S_2_O_4_) and ABTS. The mixture was incubated at room temperature for at least 16 hours in the dark. The ABTS°^+^ solution was diluted in 0.1 M PBS, pH 7.4 to absorbance at 0.700 ± 0.02 nm. The extract was added to ABTS°^+^ solution for hydrogen atom transfer (HAT). The decrease in the solution absorbance was measured at 734 nm. The TEAC value was calculated from the standard curve prepared by using a Trolox.

### Ferric reducing antioxidant power (FRAP)

The reducing power was measured according to a previous method [22]. The freshly FRAP reagent contained 0.3 M sodium acetate buffer solution (pH 3.6), 10 mM 2,4,6- tripyridyl-S-triazine (TPTZ) in 40 mM HCl and 20 mM FeCl_3_. The extract was added to FRAP solution as oxidizing reagent and incubated for 30 min at 37°C. The increase in the solution absorbance was measured at 595 nm using a spectrophotometer. FRAP value was calculated from a standard curve using FeSO_4_. FRAP value was expressed as mmol FeSO_4_/mg dried extract.

### Hydroxyl radical scavenging activity (HRSA)

Hydroxyl radical scavenging activity was measured according a previous method [22]. The absorbance was measured at 532 nm. The IC_50_ value was calculated from plots of log concentration of inhibitor concentration versus percentage inhibition curves. A trolox was used as a positive control for this study.

### Superoxide radical scavenging activity (SRSA)

Superoxide radical scavenging activity was measured according a previous method [22]. The absorbance was measured at 560 nm. The IC_50_ value was calculated from plots of log concentration of inhibitor concentration versus percentage inhibition curves. A trolox was used as a positive control for this study.

### Ferrous ion chelating power

The metal chelating power was measured according to a previously published method [20]. The decrease in the solution absorbance was measured at 522 nm. The IC_50_ value was calculated from plots of log concentration of inhibitor concentration versus percentage inhibition curves. EDTA was used as a positive control for this study.

### Statistical analysis

Data were expressed as the mean ± standard error of mean (SEM) for n = 5. The results were analyzed by one-way analysis of variance (ANOVA) and Duncan’s post hoc analysis. *p* < 0.05 was considered to be statistically significant.

## Results

### Phytochemical analysis

The content of total phenolic compounds in CTE was 53 ± 0.34 mg gallic acid equivalents/g dried extract. The content of flavonoid in CTE was 11.2 ± 0.33 mg catechin equivalents/g dried extract. Moreover, the content of total anthocyanin in CTE was 1.46 ± 0.04 mg cyanidin-3-glucoside equivalents/g dried extract.

### Antiglycation activity of CTE

**Figure 1:**
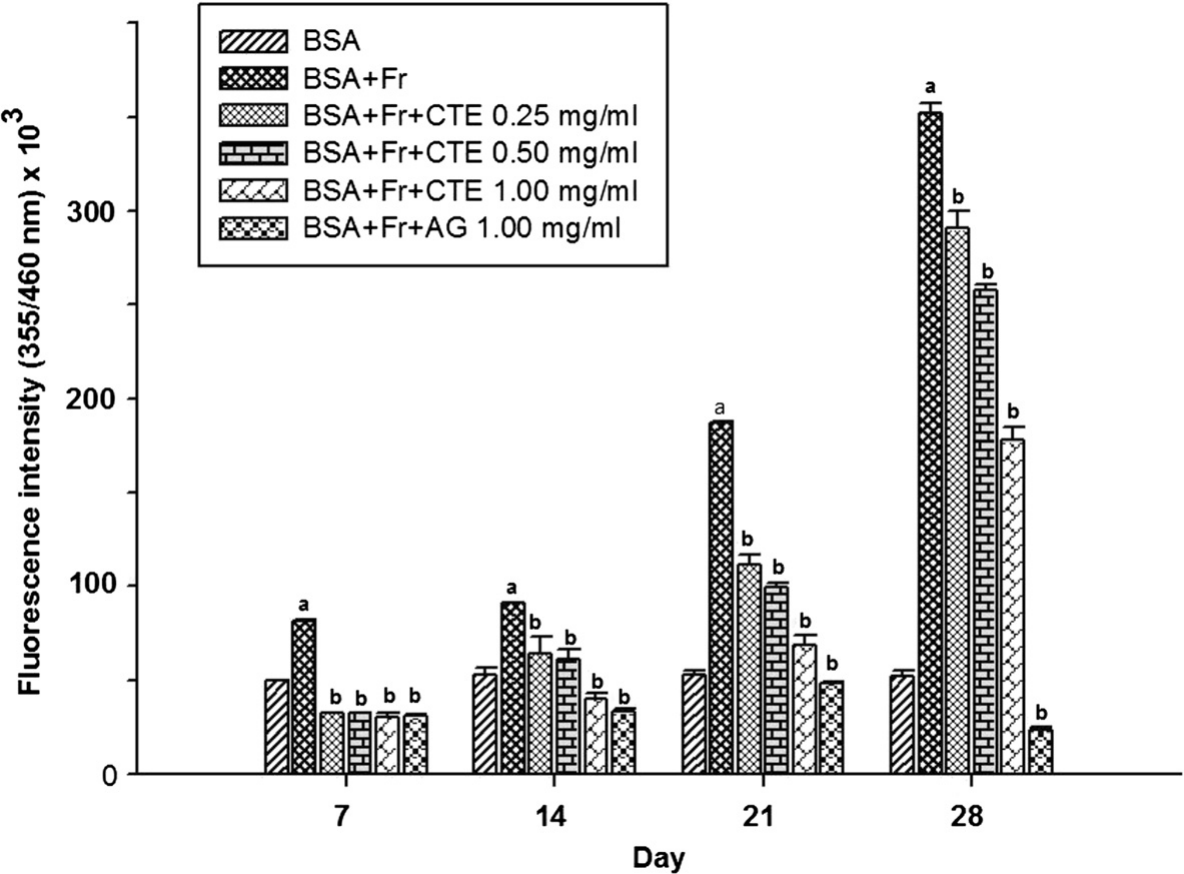
**The effects of CTE (0.25-1.00 mg/ml) and aminoguanidine (1.00 mg/ml) on fluorescent AGE formation in BSA/fructose system.** Each value represented the mean ± SEM (n = 5). ^a^ *p* < 0.05 compared to BSA, ^b^ *p* < 0.05 compared to BSA + Fr (Fructose).

**Figure 2:**
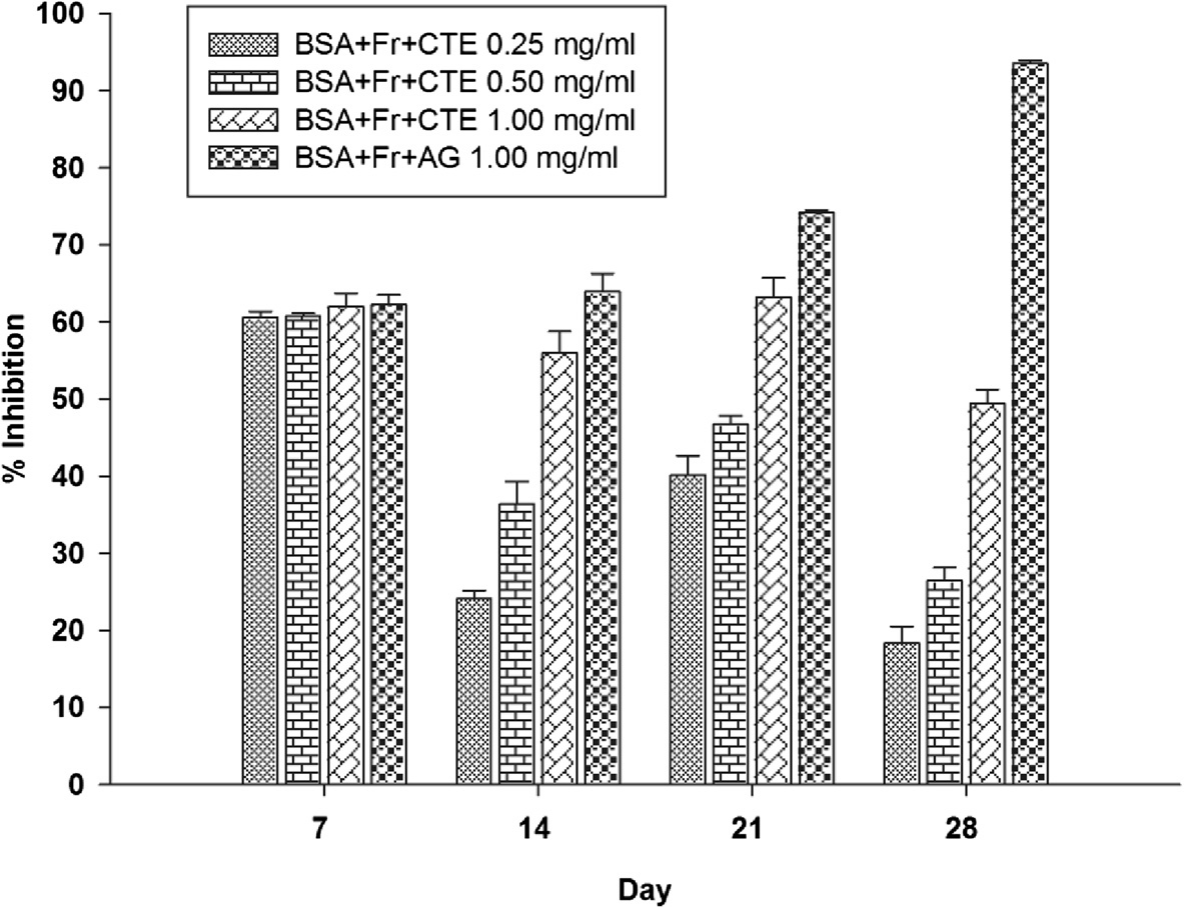
**The percentage inhibition of CTE (0.25-1.00 mg/ml) and aminoguanidine (1.00 mg/ml) on fluorescent AGE formation in BSA/fructose system.** Each value represented the mean ± SEM (n = 5).

Figure 1 shows the effects of CTE (0.25-1.00 mg/ml) on the formation of fluorescent AGEs at 7, 14, 21, and 28 days of incubation. Compared with BSA, the fluorescent intensity of glycated BSA was 1.6-fold, 1.7-fold, 3.5-fold, and 6.8-fold higher than non-glycated BSA after days 7, 14, 21, and 28, respectively. When CTE was added to the solution, the formation of AGEs was suppressed in a concentration-dependent manner during 14–28 days of incubation. At day 28, the percentage inhibition of CTE at concentration of 0.25, 0.50, and 1.00 mg/ml was 18.35%, 26.40%, and 49.40%, respectively (Figure 2). In the meantime, AG (1.00 mg/ml) inhibited AGE formation by 93.45 ± 0.45%.

### The effect of CTE on the level of fructosamine

**Figure 3:**
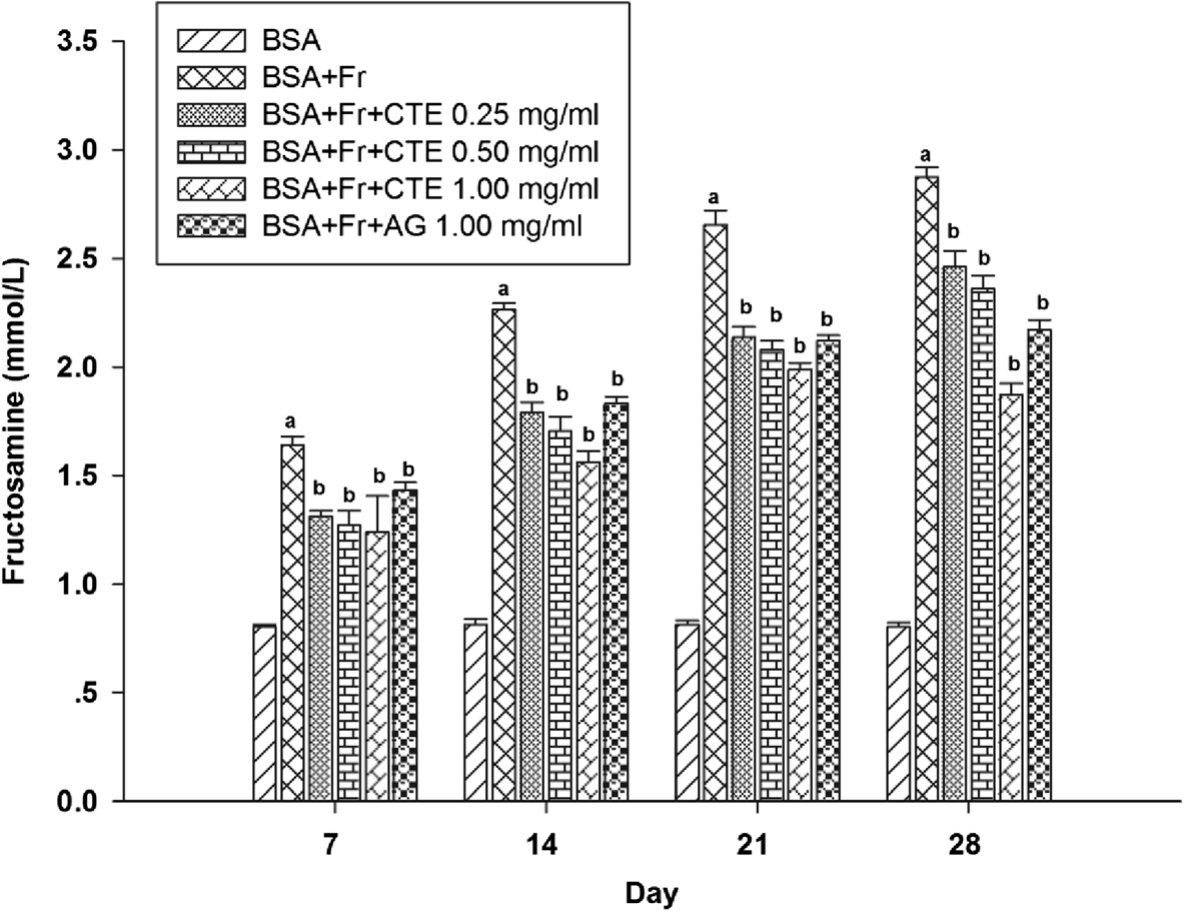
**The effects of CTE (0.25-1.00 mg/ml) and aminoguanidine (1.00 mg/ml) on the level of fructosamine in BSA/fructose system.** Each value represented the mean ± SEM (n = 5). ^a^ *p* < 0.05 compared to BSA, ^b^ *p* < 0.05 compared to BSA + Fr (Fructose).

The level of fructosamine in glycated BSA was gradually increased 2.0-fold to 3.6-fold during 28 days of incubation (Figure 3). CTE showed a significant reduction in fructosamine level in glycated BSA throughout the incubation period. At day 28, the percentage reduction of fructosamine level by CTE (0.25-1.00 mg/ml) was in the range of 14.47% to 35.66% while AG at a concentration of 1.00 mg/ml had the percentage reduction of 25.96%.

### The effect of CTE on protein carbonyl content

**Figure 4:**
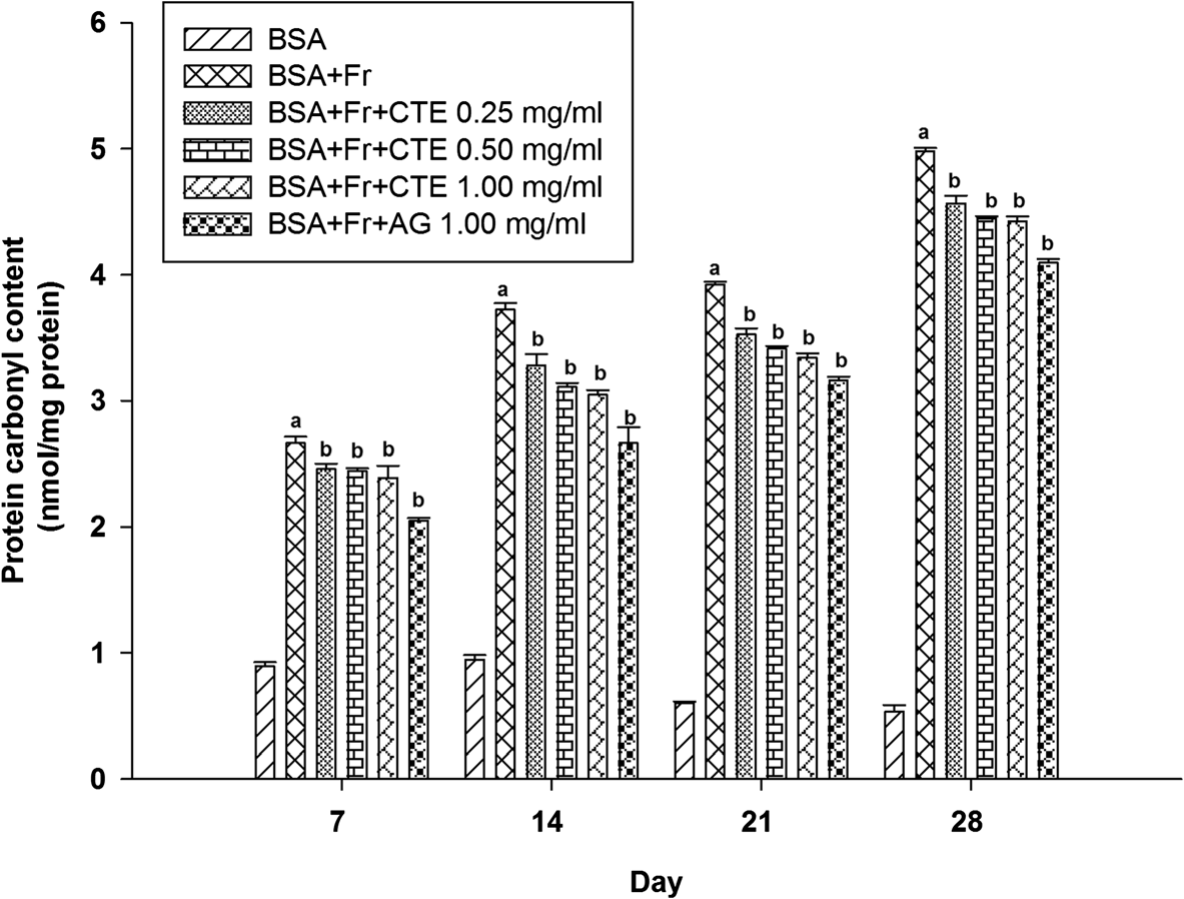
**The effects of CTE (0.25-1.00 mg/ml) and aminoguanidine (1.00 mg/ml) on the level of protein carbonyl in BSA/fructose system.** Each value represented the mean ± SEM (n = 5). ^a^ *p* < 0.05 compared to BSA, ^b^ *p* < 0.05 compared to BSA + Fr (Fructose).

Figure 4 shows the effect of CTE on protein carbonyl content represented as an oxidative modification of BSA. The carbonyl content of glycated BSA was significantly increased during the experimental period (3.0-fold to 9.4-fold increase) whereas BSA/fructose together with CTE (0.25–1.00 mg/ml) significantly attenuated an increase in the protein carbonyl content of BSA. Compared to non-glycated BSA at day 28 of incubation, the carbonyl content was reduced by CTE at concentrations of 0.25-1.00 mg/ml with the range of 8.23% to 11.34%, whereas that by AG showed a percentage of 17.71%.

### The effect of CTE on protein thiol group

**Figure 5:**
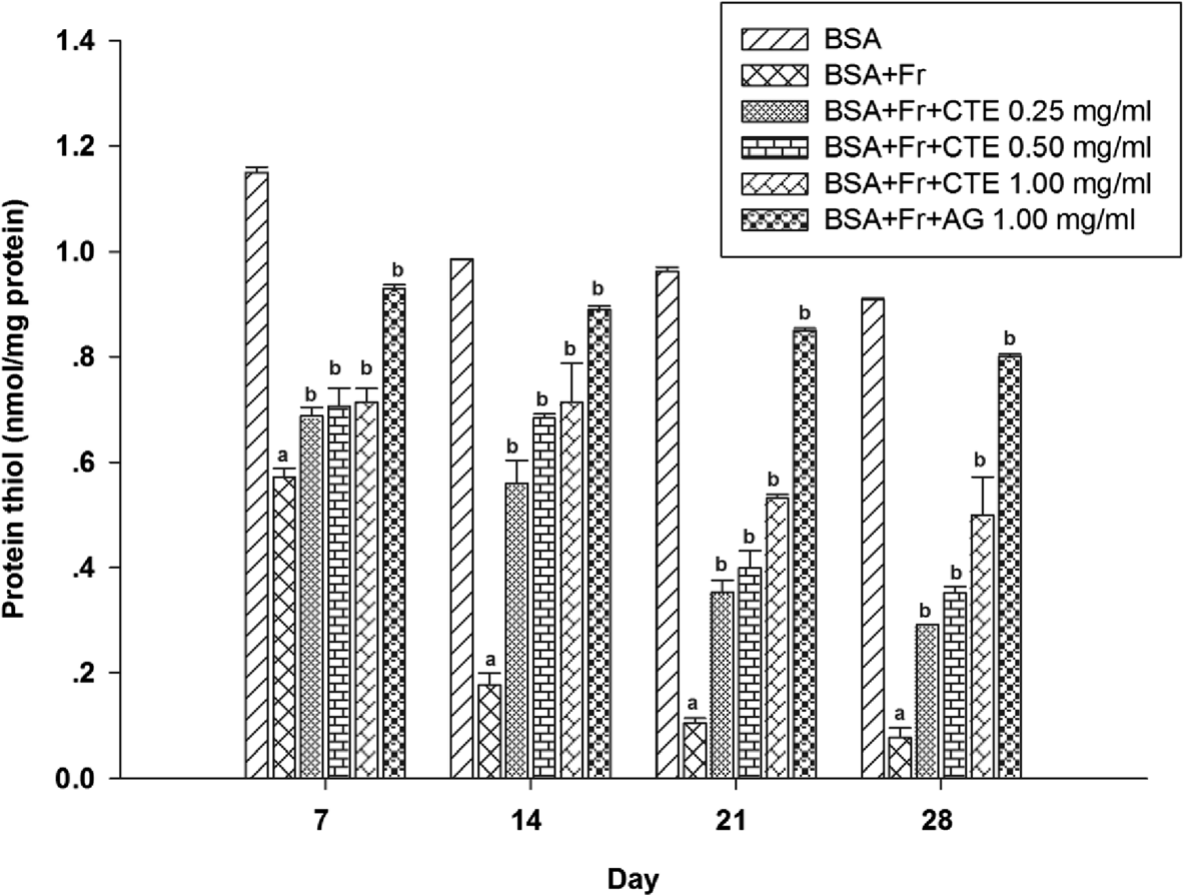
**The effects of CTE (0.25-1.00 mg/ml) and aminoguanidine (1.00 mg/ml) on the level of protein thiol group in BSA/fructose system.** Each value represented the mean ± SEM (n = 5). ^a^ *p* < 0.05 compared to BSA, ^b^ *p* < 0.05 compared to BSA + Fr (Fructose).

The structural alteration in BSA mediated by protein glycation is commonly detected by depleting the thiol group. When BSA was incubated with fructose, the level of thiol groups continuously decreased throughout the experimental period. From day 7 to 28, the percentage of depleting thiol group was in the range of 50.3%-91.6% (Figure 5). The results demonstrated that CTE at the concentration of 0.25–1.00 mg/ml significantly attenuated the depleting protein thiol group of glycated BSA during the study. At day 28, the percentage prevention of depleting thiol groups by CTE was in the range of 23.47% to 45.6% whereas AG significantly prevented the depletion of protein thiol groups by 79.57%.

### Antioxidant activity

In DPPH assay, CTE had significant radical scavenging activity with the IC_50_ value of 0.47 ± 0.01 mg/ml (Table 1). The results indicated that CTE had 235-times less potency than that observed for ascorbic acid (0.002 ± 0.001 mg/ml). According to the results from TEAC and FRAP, CTE had the ability of 0.17 ± 0.01 mg trolox equivalents/mg dried extract and 0.38 ± 0.01 mmol FeSO_4_ equivalents/mg dried extract, respectively. In the SRSA assay, the IC_50_ value of CTE was found to be 26.31 ± 4.22 mg/ml. However, CTE had 45-times less potency than trolox (0.58 ± 0.04 mg/ml). The results showed that CTE exhibited less potent antioxidant activity with FICP and HRSA.

**Table 1:** **Antioxidant activity of CTE including DPPH radical scavenging activity, TEAC, FRAP, HRSA, SRSA, and FICP**

**Antioxidant activities**						
	**DPPH**	**TEAC**	**FRAP**	**HRSA**	**SRSA**	**FICP**
CTE	0.467 ± 0.005	0.168 ± 0.001	0.379 ± 0.009	19.18 ± 3.40	26.31 ± 4.22	>10^3^
Ascorbic acid	0.002 ± 0.001	-	-	-	-	-
Trolox	-	-	-	2.03 ± 0.04	0.574 ± 0.04	-
EDTA	-	-	-	-	-	8.88 ± 1.60

Data are expressed as mean ± S.E.M, n = 3. DPPH radical scavenging activity, hydroxyl radical scavenging activity (HRSA), and superoxide radical scavenging activity (SRSA) are expressed as the IC_50_ value (mg/ml). TEAC, FRAP, and FICP are expressed as mg trolox/mg dried extract, mmol FeSO_4_/mg dried extract, and mg EDTA/mg dried extract, respectively.

## Discussion

Albumin is a target protein for the glycation reaction due to its abundance in serum [23]. Protein glycation occurs by the covalent binding of aldehyde or ketone groups of reducing sugars to the free amino groups of proteins, leading to the formation of fluorescent AGEs that can be identified by increasing fluorescent intensity [20]. The level of the Amadori products can be determined by colorimetric assay. Our results in this experiment showed that CTE effectively inhibited fructose-induced fluorescent AGE formation in a concentration-dependent manner. In addition, the inhibitory effect of CTE consequently suppressed the formation of fructosamine (Amadori adducts) and AGEs. Previous studies have shown the ability of phenolic-enriched plant extracts against fructose-induced protein glycation [24,25]. For example, pomelo extract (0.25 – 1 mg/ml) inhibited the overall formation of AGEs approximately 50-86% [26]. At concentrations of 1 mg/ml, Beijing grass, pennywort, gingko, Cat’s Whiskers and grape seed containing phenolic compounds and flavonoids had the percentage inhibition of protein glycation ranging 17-41% at week 4 of experiments [27]. *Mesona chinensis* Benth (Chinese Mesona), most widely consumed as an herbal beverage and a gelatin-type dessert, also showed the percentage inhibition of 39.60-59.42% with concentration of 0.25-1.00 mg/mL at week 4 of incubation [28]. Our findings suggest that CTE is a moderate antiglycating agent in comparison with other phenolic-enriched extracts.

Abundant evidence exists that an excessive production of reactive oxygen species (ROS) and reactive nitrogen species are generated during glycation and glycoxidation [3-5] The production of ROS causes the oxidation of amino acid residues of protein to form a carbonyl derivative, which diminishes the oxidative defence of protein by eliminating the thiol groups [29,30]. In the present study, the protein oxidation was observed by increasing protein carbonyl content and depleting protein the thiol group of BSA. Conversely, the reduction of protein carbonyl content and oxidation of thiol group of BSA/fructose system was affected by CTE. Evidence supports that the formation of AGEs could generate free radicals and highly reactive intermediates in the early stages of glycation [31]. Moreover, the Amadori products subsequently degrade into α-dicarbonyl compounds such as methylglyoxal, glyoxal, and deoxyglucosones. These compounds are more reactive than the reducing sugars with respect to their ability to react with amino groups of proteins and consequently generate the cross-linked methylglyoxal dialkylimine radical cation and the enediol radical anion of methylglyoxal, which leads to the formation of AGEs [32]. In addition, the methylglyoxal anions directly react with molecular oxygen to generate the superoxide anion radicals whereas hydroxyl radicals can be generated during the reaction of methylglyoxal with lysine in the presence of metal ions (Fe^3+^ and Cu^2+^) [32]. When ROS is increased from protein glycation reaction, antioxidant enzymes (superoxidae dismutase, catalase, and glutathione reductase) primarily account for intracellular defense while non-enzyme antioxidants (vitamin C and E) help protect various components against oxidative damage in plasma [33]. Interestingly, it has been shown that free radical scavengers from natural products can act as non-enzyme antioxidants to prevent against reducing sugar-induced glycation and oxidative modification to protein [4]. Therefore, various antioxidant activity methods have been used to investigate the free radical scavenging ability of CTE. The findings indicate that CTE exhibits the ability to scavenge different forms of free radicals, thus suggesting that its potential use in preventing formation of AGEs and oxidative modification to protein is possibly as a result of free radical scavenging ability.

Phytochemical compounds including polyphenolics, flavonoids and anthocyanins, present in plant-based foods, provide beneficial effects on free radical scavenging properties [34-36]. The phytochemical analysis of *Clitoria ternatea* flower petal extract revealed the presence of bioactive compounds such as delphinidin-3,5-glucoside, delphinidin-3β-glucoside, malvidin-3β-glucoside, kaemphferol, *p*-coumaric acid and six major ternatins (ternatins A1, A2, B1, B2, D1 and D2) [37]. The previous findings demonstrated in the phytochemical compounds possibly contributing to anti-glycation and antioxidant activity, the amount of phenolics and flavonoids in plant-based foods is highly related to their activity [14,15,38]. In the view of the above-mentioned, the probable mechanism by which CTE exerts antiglycation and antioxidant activity may be in association with the phytochemical compounds in the extract. Other mechanisms of antiglycation, particularly for inhibiting the formation of late-stage Amadori products, breaking the cross-linking structures in the intracellular formed AGEs, and blocking the receptor for advanced glycation end products (RAGEs) have been proposed [4]. Further comprehensive studies of CTE are required to clarify the antiglycation mechanisms described above.

## Conclusions

In the present work, CTE prevents fructose-induced protein glycation and oxidation-dependent damages to protein. It also exhibits an excellent scavenging ability for different forms of free radicals. Antiglycation and antioxidant activity of CTE may be responsible for their usefulness in the management and prevention of AGE-mediated diabetic complication. Further studies are required to investigate the antiglycative effects of CTE on diabetic rats.

## Abbreviations

AGEs: Advanced glycation end-products
CTE: *Clitoria ternatea* extract
BSA: Bovine serum albumin
AG: Aminoguanidine
DPPH: 1,1-diphenyl 2-picrylhydrazyl radical scavenging activity
TEAC: Trolox equivalent antioxidant capacity
FRAP: Ferric reducing antioxidant power
HRSA: Hydroxyl radical scavenging activity
SRSA: Superoxide radical scavenging activity
FICP: Ferrous ion chelating power

## References

[1] Giacco F, Brownlee M (2010). Oxidative stress and diabetic complications. Circ Res.

[2] Stitt AW (2001). Advanced glycation: an important pathological event in diabetic and age related ocular disease. Br J Ophthalmol.

[3] Singh R, Barden A, Mori T, Beilin L (2001). Advanced glycation end-products: a review. Diabetologia.

[4] Wu CH, Huang SM, Lin JA, Yen GC (2011). Inhibition of advanced glycation endproduct formation by foodstuffs. Food Funct.

[5] Ahmed N (2005). Advanced glycation endproducts-role in pathology of diabetic complications. Diabetes Res Clin Pract.

[6] Goh SY, Cooper ME (2008). Clinical review: the role of advanced glycation end products in progression and complications of diabetes. J Clin Endocrinol Metab.

[7] Basta G, Schmidt AM, De Caterina R (2004). Advanced glycation end products and vascular inflammation: implications for accelerated atherosclerosis in diabetes. Cardiovasc Res.

[8] Ramasamy R, Vannucci SJ, Yan SS, Herold K, Yan SF, Schmidt AM (2005). Advanced glycation end products and RAGE: a common thread in aging, diabetes, neurodegeneration, and inflammation. Glycobiology.

[9] Bell RC, Carlson JC, Storr KC, Herbert K, Sivak J (2000). High-fructose feeding of streptozotocin-diabetic rats is associated with increased cataract formation and increased oxidative stress in the kidney. Br J Nutr.

[10] Tappy L, Le KA (2010). Metabolic effects of fructose and the worldwide increase in obesity. Physiol Rev.

[11] Suárez G, Maturana J, Oronsky AL, Raventós-Suárez C (1991). Fructose-induced fluorescence generation of reductively methylated glycated bovine serum albumin: evidence for nonenzymatic glycation of Amadori adducts. Biochim Biophys Acta.

[12] Thornalley PJ (2003). Use of aminoguanidine (Pimagedine) to prevent the formation of advanced glycation endproducts. Arch Biochem Biophys.

[13] Friedman EA (2010). Evolving pandemic diabetic nephropathy. Rambam Maimonides Med J.

[14] Ramkissoon JS, Mahomoodally MF, Ahmed N, Subratty AH (2013). Antioxidant and anti-glycation activities correlates with phenolic composition of tropical medicinal herbs. Asian Pac J Trop Med.

[15] Kusirisin W, Srichairatanakool S, Lerttrakarnnon P, Lailerd N, Suttajit M, Jaikang C (2009). Antioxidative activity, polyphenolic content and anti-glycation effect of some Thai medicinal plants traditionally used in diabetic patients. Med Chem.

[16] Mukherjee PK, Kumar V, Kumar NS, Heinrich M (2008). The Ayurvedic medicine *Clitoria ternate*a-from traditional use to scientific assessment. J Ethnopharmacol.

[17] Gupta GK, Chahal J, Bhatia M (2010). *Clitoria ternatea* (L.): old and new aspects. J Pharm Res.

[18] Soundrapandian C, Datta S, Sa B (2007). Drug-eluting implants for osteomyelitis. Crit Rev Ther Drug Carrier Syst.

[19] Adisakwattana S, Ruengsamran T, Kampa P, Sompong W (2012). In vitro inhibitory effects of plant-based foods and their combinations on intestinal α-glucosidase and pancreatic α-amylase. BMC Complement Altern Med.

[20] Jariyapamornkoon N, Yibchok-anun S, Adisakwattana S (2013). Inhibition of advanced glycation end products by red grape skin extract and its antioxidant activity. BMC Complement Altern Med.

[21] Adisakwattana S, Sompong W, Meeprom A, Ngamukote S, Yibchok-Anun S (2012). Cinnamic acid and its derivatives inhibit fructose-mediated protein glycation. Int J Mol Sci.

[22] Mäkynen K, Jitsaardkul S, Tachasamran P, Sakai N, Puranachoti S, Nirojsinlapachai N (2013). Cultivar variations in antioxidant and antihyperlipidemic properties of pomelo pulp (*Citrus grandis* [L.] Osbeck) in Thailand. Food Chem.

[23] Wautier J, Guillausseau P (2001). Advanced glycation end products, their receptors and diabetic angiopathy. Diabetes Metab.

[24] Dorsey PG, Greenspan P (2014). Inhibition of nonenzymatic protein glycation by pomegranate and other fruit juices. J Med Food.

[25] Dearlove RP, Greenspan P, Hartle DK, Swanson RB, Hargrove JL (2008). Inhibition of protein glycation by extracts of culinary herbs and spices. J Med Food.

[26] Caengprasath N, Ngamukote S, Mäkynen K, Adisakwattana S (2013). The protective effects of pomelo extract (*Citrus Grandis* L. Osbeck) against fructose-mediated protein oxidation and glycation. EXCLI J.

[27] Adisakwattana S, Jiphimai P, Prutanopajai P, Chanathong B, Sapwarobol S, Ariyapitipan T (2010). Evaluation of alpha-glucosidase, alpha-amylase and protein glycation inhibitory activities of edible plants. Int J Food Sci Nutr.

[28] Adisakwattana S, Thilavech T, Chusak C (2014). *Mesona Chinensis* Benth extract prevents AGE formation and protein oxidation against fructose-induced protein glycation in vitro. BMC Complement Altern Med.

[29] Roche M, Rondeau P, Singh NR, Tarnus E, Bourdon E (2008). The antioxidant properties of serum albumin. FEBS Lett.

[30] Chesne S, Rondeau P, Armenta S, Bourdon E (2006). Effects of oxidative modifications induced by the glycation of bovine serum albumin on its structure and on cultured adipose cells. Biochimie.

[31] Smith PR, Thornalley PJ (1992). Mechanism of the degradation of non‐enzymatically glycated proteins under physiological conditions. Eur J Biochem.

[32] Kang JH (2003). Oxidative damage of DNA induced by methylglyoxal in vitro. Toxicol Lett.

[33] Szaleczky E, Prechl J, Fehér J, Somogyi A (1999). Alterations in enzymatic antioxidant defence in diabetes mellitus-a rational approach. Postgrad Med J.

[34] Rice-Evans C, Miller N, Paganga G (1997). Antioxidant properties of phenolic compounds. Trends Plant Sci.

[35] Pietta PG (2000). Flavonoids as antioxidants. J Nat Prod.

[36] Einbond LS, Reynertson KA, Luo X-D, Basile MJ, Kennelly EJ (2004). Anthocyanin antioxidants from edible fruits. Food Chem.

[37] Terahara N, Oda M, Matsui T, Osajima Y, Saito N, Toki K (1996). Five new anthocyanins, ternatins A3, B4, B3, B2, and D2, from *Clitoria ternatea* flowers. J Nat Prod.

[38] Ho SC, Wu SP, Lin SM, Tang YL (2010). Comparison of anti-glycation capacities of several herbal infusions with that of green tea. Food Chem.

